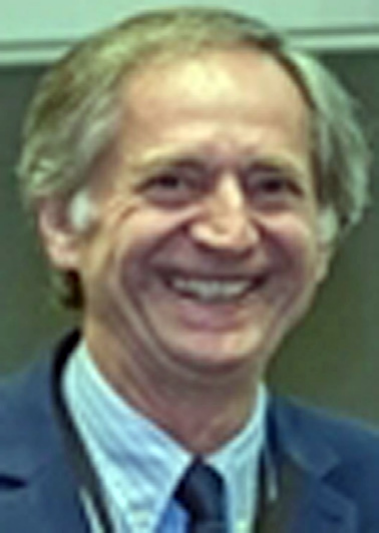# Enhancing the Activity of Drugs by Conjugation to Organometallic Fragments

**DOI:** 10.1002/chem.201904699

**Published:** 2020-05-26

**Authors:** Prinessa Chellan, Peter J. Sadler

**Affiliations:** ^1^ Department of Chemistry and Polymer Science Stellenbosch University 7600 Matieland, Western Cape South Africa; ^2^ Department of Chemistry University of Warwick Coventry CV4 7AL UK

**Keywords:** anticancer complexes, antimicrobial complexes, bioinorganic chemistry, bioorganometallic chemistry, coordination chemistry

## Abstract

Resistance to chemotherapy is a current clinical problem, especially in the treatment of microbial infections and cancer. One strategy to overcome this is to make new derivatives of existing drugs by conjugation to organometallic fragments, either by an appropriate linker, or by direct coordination of the drug to a metal. We illustrate this with examples of conjugated organometallic metallocene sandwich and half‐sandwich complexes, Ru^II^ and Os^II^ arene, and Rh^III^ and Ir^III^ cyclopentadienyl half‐sandwich complexes. Ferrocene conjugates are particularly promising. The ferrocene–chloroquine conjugate ferroquine is in clinical trials for malaria treatment, and a ferrocene‐tamoxifen derivative (a ferrocifen) seems likely to enter anticancer trails soon. Several other examples illustrate that organometallic conjugation can restore the activity of drugs to which resistance has developed.

## Graphical abstract

**There is an urgent clinical need** for new agents to treat microbial infections and cancer, especially to combat resistance to current drugs. The successful strategy of conjugating existing drugs to organometallic fragments is illustrated with examples of conjugated organometallic metallocene sandwich and half‐sandwich complexes, especially ferrocene, Ru^II^ and Os^II^ arene, and Rh^III^ and Ir^III^ cyclopentadienyl complexes.

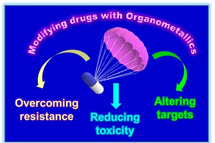

## Introduction

Resistance to chemotherapy is a major clinical problem. This arises either from inherent lack of sensitivity to current drugs or induced resistance on repeated treatment. In particular, resistance is a problem for the treatment of cancer and microbial diseases. One approach to overcoming this problem is to design new drugs with novel mechanisms of action. Here we focus on the potential for discovery of novel drugs by incorporating organometallic fragments, so extending designs to the wider periodic table beyond purely organic drugs.^1^ In particular we highlight the use of organometallic fragments to re‐activate organic drugs towards which resistance has developed.

In general, metal complexes are now finding wider applications in medicine, particularly against cancer, but the basis for use has often been empirical without detailed understanding of structure–activity relationships, or identification of their target sites and mechanisms of action. However, times are changing.

**Figure 1:**
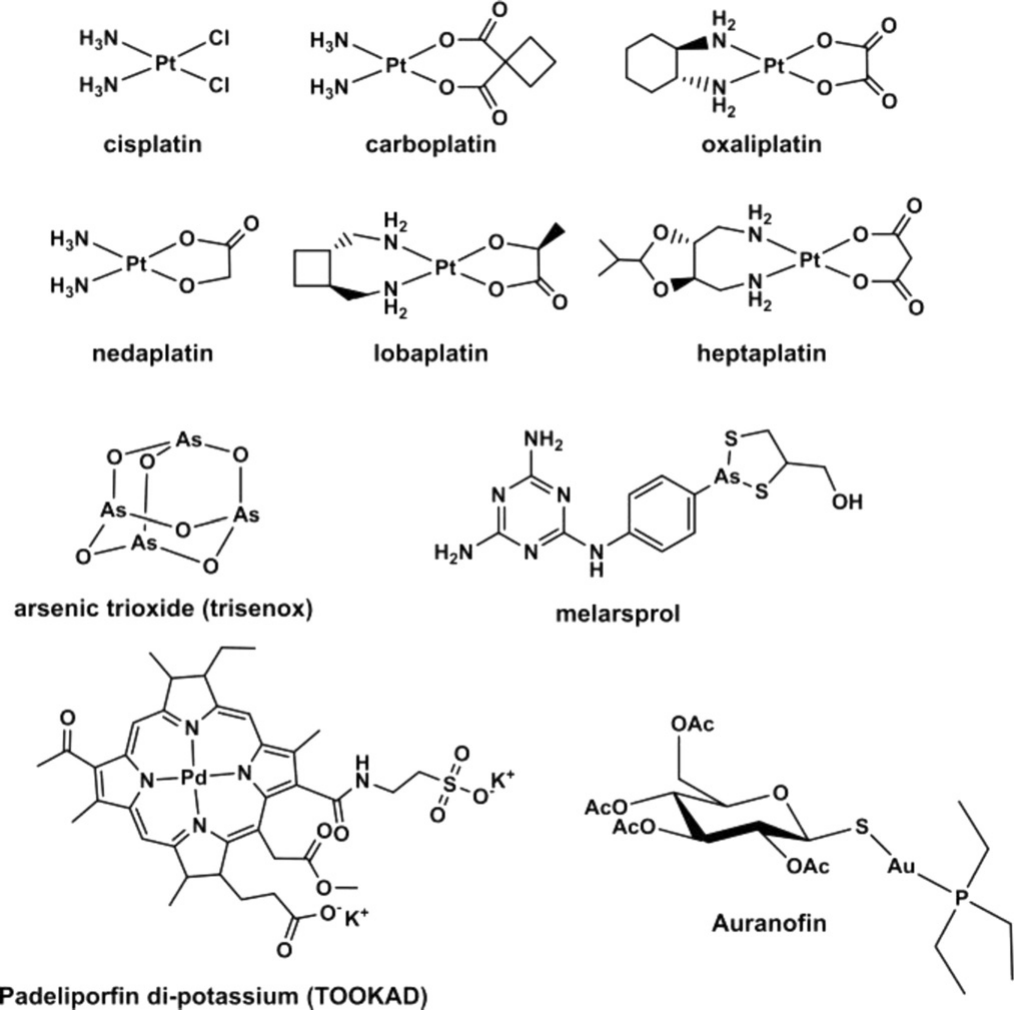
Examples of some metal complexes which are drugs.

The major development of platinum‐based anticancer drugs has helped to establish metal complexes as viable treatments.^2^, ^3^, ^4^, ^5^, ^6^, ^7^, ^8^, ^9^ The platinum‐containing drug cisplatin (Figure 1) and later generation analogues carboplatin and oxaliplatin are now widely used to treat various cancers.^2^ More recently, nedaplatin, lobaplatin and heptaplatin have been approved for chemotherapy in certain Asian countries.^10^ This family of drugs mostly target DNA, causing structural changes through Pt–DNA adducts which lead to apoptosis and cancer cell death.^2^, ^11^ However, small changes in structure can dramatically change the target, for example, oxaliplatin targets ribosome biogenesis not DNA.^12^ Other metal complexes have long been used in treatment, including arsenic trioxide, marketed as Trisenox, for the blood cancer acute promyelocytic leukaemia,^13^, ^14^, ^15^ melasoprol, an organoarsenical for trypanosome infections in Africa,^16^ and Bi^III^ antimicrobial/antiulcer compounds such as colloidal bismuth subcitrate (CBS, De‐Nol) and ranitidine bismuth citrate (RBC, Pyrlorid).^17^ Padeliporfin di‐potassium (TOOKAD) was recently registered for use as a vascular targeted photodynamic therapeutic agent against prostate cancer.^18^ The gold complex, auranofin (Figure 1), is prescribed for rheumatoid arthritis treatment; classified as a disease‐modifying antirheumatic which slows progression through suppression of inflammation and encouraging cell‐mediated immunity.^19^ Recently it has entered clinical trials as a broad‐spectrum antiparasitic drug.^20^

**Figure 2:**
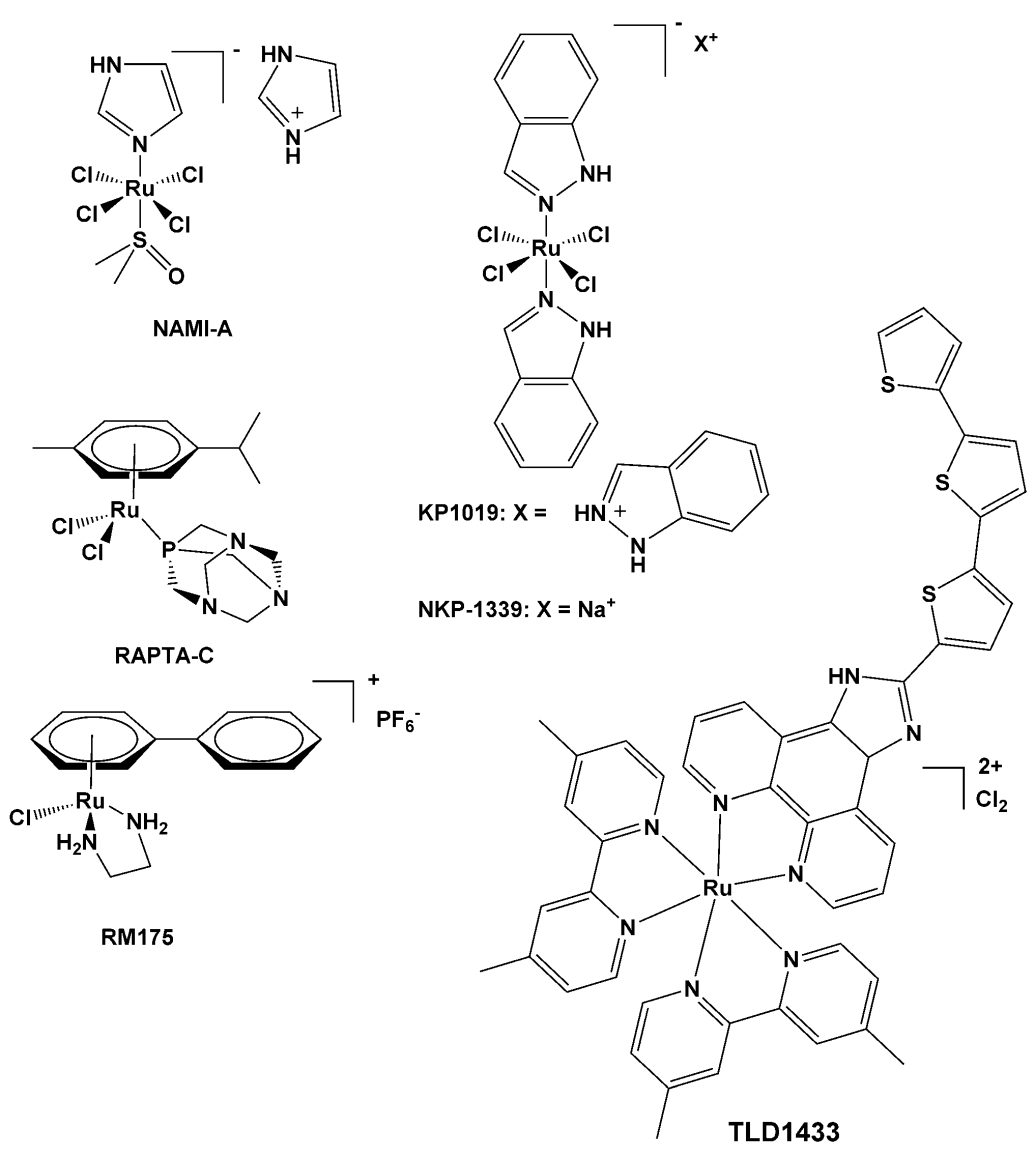
Early Ru^III^ and Ru^II^ complexes studied for anticancer activity. Ru^III^ complexes are thought to be activated by in vivo reduction to Ru^II^.

There are increasing numbers of novel inorganic and organometallic complexes under development as therapeutic agents with several currently in clinical trials or already approved for clinical use.^2^, ^21^ In particular, compounds with metals from the platinum group metals (PGM)—ruthenium, rhodium, palladium, osmium, iridium, and platinum—have risen to prominence in the search for new inorganic drugs, for example, highly water soluble Ru^III^ chlorido‐DMSO complexes like NAMI‐A (Figure 2), first studied by Mestroni, Alessio, Sava et al.^22^, ^23^, ^24^ This drug is an anti‐metastatic agent, either inhibiting metastasis formation^25^ or reducing the size of metastases.^26^ It completed phase I clinical trials.^27^, ^28^, ^29^, ^30^ However, in a phase I/II combination with gemcitabine in patients with non‐small cell lung cancer,^31^ it did not show improvement over gemcitabine alone.^32^

The antineoplastic agent KP‐1019 developed by Keppler and co‐workers has completed phase I trials;^30^, ^33^, ^34^, ^35^, ^36^, ^37^, ^38^, ^39^ its sodium salt, NKP‐1339 is in further clinical development.^40^ Ruthenium(II) arene complexes such as RM175^38^, ^41^ and RAPTA‐C^42^, ^43^, ^44^ (Figure 2) have stimulated interest in the design of half‐sandwich organometallic anticancer complexes, as well as possible applications for treatment of malaria and tuberculosis.^9^, ^45^, ^46^, ^47^ TLD1433 is a polypyridyl ruthenium complex and first example of a Ru^II^‐based photosensitizer to enter human clinical trials.^48^

The use of organometallic fragments in the redesign and repurposing of organic drugs shows promise for reactivation, combatting resistance, and increasing potency. Recently the power of ferrocene labelling, for example, has been widely demonstrated. Ferrocene is an organometallic compound containing Fe^II^ and two cyclopentadienyl ligands in a rigid sandwich structure. This unit is stable in physiological media and has a lipophilic nature that allows it to traverse cell membranes effectively.^49^, ^50^, ^51^ It can also be derivatised with relative ease to allow incorporation into a range of drug scaffolds.^51^, ^52^, ^53^ In an early example (1975), the phenyl or heteroaromatic groups of penicillin or cephalosporin were replaced by a ferrocene moiety, giving new highly active antibiotics and potent β‐lactamase inhibitors.^54^

Here we discuss some selected examples of organometallic conjugates of clinical drugs, categorised by the clinical application of the ‘parent’ organic drug. Other relevant reviews of bioorganometallic complexes, focus either on a specific disease, disease subset (such as parasitic), or a structural element.^55^, ^56^, ^57^, ^58^

We have restricted our discussion to derivatives where the structure of the clinical drug remains largely intact and with applications against a variety of diseases and conditions.

## Derivatives of Cancer Drugs

**Figure 3:**
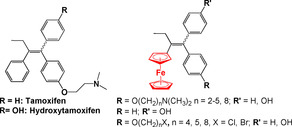
Tamoxifen and the early ferrocifen derivatives.^57^, ^59^, ^60^, ^61^

**Figure 4:**
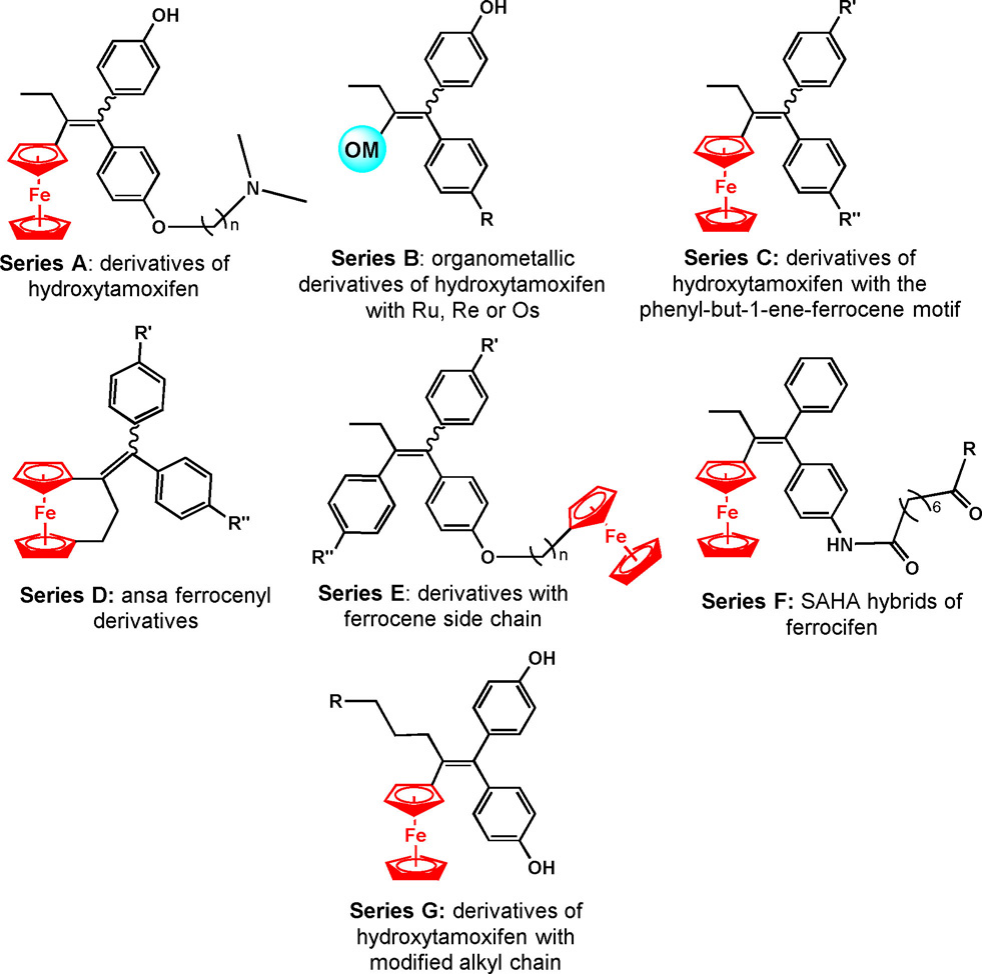
Some of the libraries generated from the initial ferrocifen complexes.^57^ R, R’ and R’’=alkyl, hydroxy, alkylamine or amino groups.

Tamoxifen and its active metabolite hydroxytamoxifen (Figure 3) are breast cancer chemotherapeutics. Ferrocenyl derivatives of these drugs, aptly named ferrocifens, have been extensively studied by Jaouen, Vessiéres, Top and co‐workers since the mid‐1990s, starting with the ferrocenyl complexes shown in Figure 3.^59^, ^60^, ^61^ These early complexes, are selective estrogen receptor modulators (SERMs), and exhibit good inhibitory effects on both MCF‐7 (hormone dependent) and MDA‐MB‐231 (hormone independent) breast cancer cells in contrast to their organic precursor, hydroxytamoxifen, which is active only against the MCF‐7 cell line. The discovery of this dual effect prompted the synthesis of several different libraries of ferrocifen‐type complexes (Figure 4).^57^

Each series has been extensively studied for their effects on MCF‐7 breast cancer cells.^57^ In vitro antiproliferative studies on the breast cancer cell lines MDA‐MB‐231 and MCF‐7, the human prostate PC‐3 cancer cell line, and healthy cell lines, showed that these compounds have higher selectivity for cancer cells versus normal cells.^57^ Ferrocene itself is less active compared to the ferrocifen derivatives and the presence of the ferrocenyl‐double bond‐phenol motif (Series C, where R’, R’’=H, OH, or NH_2_, Figure 4) is essential for high antiproliferative effects.^57^, ^62^, ^63^ When the ferrocene unit is incorporated at the end of the sidechains as in Series E, a reduction in activity is observed relative to Series C.^63^ Series D, ansa ferrocenyl analogues, do not contain a dimethylaminoalkoxy group like the original ferrocifen complexes (Series A) and were more active than these open‐chain homologues.^64^ Comparison of Series C (acyclic) and Series D (ansa) also showed that the closed‐ring analogues were more active; the complex with R’ and R’’=OH exhibited an IC_50_ value of 0.09 μm for resistant MDA‐MB‐231 cells. It was seven times more potent than its acyclic derivative. A similar increase in activity against the resistant breast cancer cell line was obtained for the ansa complex where R’=NH_2_ and R’’=OH. Series F, containing suberoylanilide hydroxyamic acid (SAHA) or phenylsuberamide (PSA), was more active (IC_50_ values between 0.5–0.7 μm) compared to ferrocifen‐OH (IC_50_=2.6 μm), tamoxifen SAHA (IC_50_=8.6 μm) and tamoxifen PSA (IC_50_=25.2 μm) derivatives.^65^ SAHA inhibits histone deacetylases, enzymes involved in gene expression.

The osmium and ruthenium metallocene derivatives of hydroxytamoxifen (Series B) are not as active as their iron homologues (Series A).^66^ A detailed account of the synthesis, inhibitory activities and mechanistic studies on ferrocifens has been published.^57^ Oxidation of ferrocifens in cells to active quinone methide metabolites appears to play a key role in their mechanism of action.^57^

In Series G, where the ethyl group was replaced with a longer ester or hydroxy‐modified alkyl chain,^[[67]^ the complexes exhibited low micromolar activity against MDA‐MB‐231 cancer cells.

**Figure 5:**
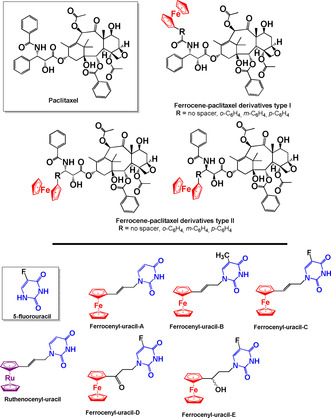
Structures of paclitaxel, 5‐fluorouracil, ferrocene‐paclitaxel derivatives type I and II, and metallocene‐uracil derivatives.^68^, ^72^

Paclitaxel (Figure 5) is a diterpene that belongs to the group of antimitotic agents known as taxanes. Marketed as Taxol or Onxal, it is an intravenous anticancer drug used in treatment of ovarian and breast carcinomas and Kaposi's sarcoma. Paclitaxel exerts its chemotherapeutic effect through promotion of tubulin polymerisation and stabilisation and impairment of microtubules causing mitotic arrest and apoptosis of cancer cells.^68^, ^69^, ^70^ Ferrocenyl derivatives of paclitaxel have been studied for antiproliferative activity in the human tumour cell lines A549 (alveolar basal epithelial cell adenocarcinoma), Colo 205 (colorectal adenocarcinoma), HCT116 (colorectal adenocarcinoma), Hep G2 (hepatocellular carcinoma), MCF7 (breast adenocarcinoma) and SW620 (colorectal adenocarcinoma) by Rychlik and co‐workers.^68^ Efficacy was dependent on the position of the ferrocene moiety, and the *N*‐benzoyl substituted derivatives (ferrocene‐paclitaxel derivatives type **I**, Figure 5) showed the most promising activity. The spatial positioning of ferrocene greatly affected the activity; for the type **I** derivatives the best activity was observed when there was no aromatic spacer (IC_50_=0.005–0.0015 μm), against W620, A549, Colo 205, HCT116, Hep G2 and MCF7 cells. Lower activities were noted for complexes containing the ferrocene substituted *N*‐benzoyl group in the order *m*‐substituted > *p*‐substituted > *o*‐substituted. Substitution of the 3’‐phenyl group in the phenylisoserine side chain of paclitaxel with ferrocenyl groups (ferrocene‐paclitaxel derivatives type II) did not yield any notable activity. These complexes were also assayed for activity in a panel of five multidrug‐resistant cell lines derived from SW620 and characterized by overexpression of various ATP‐Binding Cassette (ABC) proteins, namely ABCG2 (SW620C line), ABCC1 (SW620M and SW620E lines) and ABCB1 (SW620D, SW620E, and SW620V lines). ABC proteins are a group of transporter proteins responsible for drug resistance and reduced bioavailability of drugs by efflux of the drug from cells.^71^ In particular, the expression of the ABCB1 efflux protein adversely affects the activity of taxanes in cancer cells. Complexes of type **I** showed good inhibition of the ABCB1‐expressing cell lines, SW620D, SW620E and SW620V. Determination of the induction of tubulin polymerization and ROS generation by these compounds led to the conclusion that there is no general mechanism for these ferrocenyl‐paclitaxel complexes. Compounds showing robust pro‐oxidative properties exhibited lower anticancer activities and complexes that were good polymerization inducers showed either high or low anticancer activity. Nevertheless, these ferrocenyl‐paclitaxel complexes are a good starting point for further development of ferrocene taxane anticancer drugs and it is likely that one such complex will reach clinical trials.

5‐Fluorouracil is administered intravenously to treat a variety of cancers including, colon, stomach and breast, and is used topically for some skin cancers. Moderate inhibition was observed for ferrocene‐uracil derivatives (Figure 5) in the MCF‐7 breast cancer cell line.^72^ Structure‐activity analysis showed that introduction of methyl or fluoro substituents on the uracil in **Ferrocenyl‐uracil‐A** activated the antiproliferative effects, with **Ferrocenyl‐uracil‐B** being the most potent (IC_50_ 23.8 μm). The **ruthenocenyl‐uracil** complex did not show any appreciable activity and measurement of ruthenium levels in HT‐29 cells after exposure to a 100 μm concentration of the complex revealed that Ru is taken up by the cells over time but poorly, which may account for the low activity observed. These complexes were not screened in a resistant cancer cell line so their selectivity cannot be assessed.

Antibacterial screening of **ferrocenyl‐uracils C‐E** on Gram‐positive *Staphylococcus aureus* (*SA*) and *Staphylococcus epidermidis* (*SE*) bacterial strains revealed moderate to good activity for these complexes. Their MIC values were the same for methicillin‐sensitive (MSSA), methicillin‐resistant (MRSA), and vancomycin‐resistant (VRSA) strains, suggesting no selectivity towards resistant bacteria. **Ferrocenyl‐uracil‐D** was the most promising, with MIC values of 28 μg mL^−1^ on the SA strains and 16 μg mL^−1^ on the SE strain. 5‐Fluorouracil was not tested at the same time as the ferrocenyl complexes.

## Derivatives of Antibiotics

**Figure 6:**
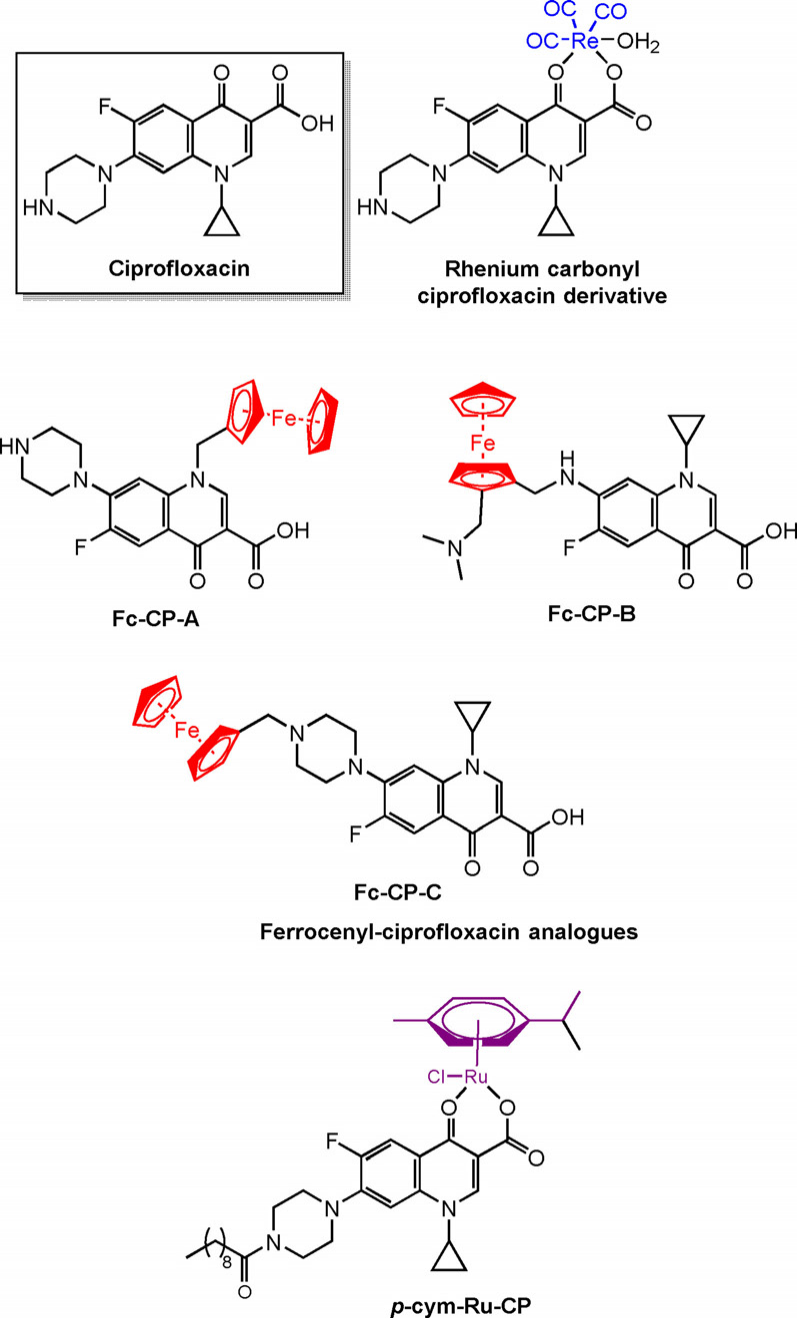
Organometallic derivatives of ciprofloxacin.^75^, ^76^

Fluoroquinolones (e.g., ciprofloxacin, **CP**, Figure 6) are a class of antibiotics, used especially for treatment of respiratory and urinary tract infections. **CP** is a broad spectrum agent also used as a prophylactic for neutropenia and in veterinary medicine.^5^ **CP** is active against both chloroquine‐sensitive and chloroquine‐resistant *P. falciparum* strains, which led to studies of its effect on patients with acute falciparum malaria.^73^, ^74^ However, the drug was less potent than chloroquine; displaying delayed inhibitory effects, thus limiting its potential use as an antimalarial. As a result of success with ferroquine (vide infra), Biot and co‐workers extended their search for organometallic conjugates to ferrocenyl‐**CP** derivatives.^5^, ^75^ An enhancement of efficacy was reported when compared to **CP** and the antimalarial drugs, chloroquine, quinine and artesunate, displaying IC_50_ values between 0.8–3.9 μm in CQ‐resistant (W2) and CQ‐sensitive (3D7) parasite strains. All the complexes were active against both W2 and 3D7, suggesting that these ferrocenyl‐**CP** derivatives are either not susceptible to the same resistance mechanisms as CQ, or that they have different molecular targets. **Complex Fc‐CP‐A** was the most potent, with IC_50_ values of 1.0 and 0.8 μm against 3D7 and W2, respectively, 20 times greater (3D7) and 460 times greater (W2) than CQ, and comparable to artesunate.^75^

The rhenium carbonyl derivative of **CP** (Figure 6) has activity similar to free **CP** towards Gram positive and Gram negative bacteria.^76^ The Ru^II^ arene complex ***p*** **‐cy‐Ru‐Cp** containing chelated **CP**, retained moderate anti‐microbial activity in a clinical *E. coli* isolate that is resistant to first, second and third generation β‐lactam antibiotics.^77^

**Figure 7:**
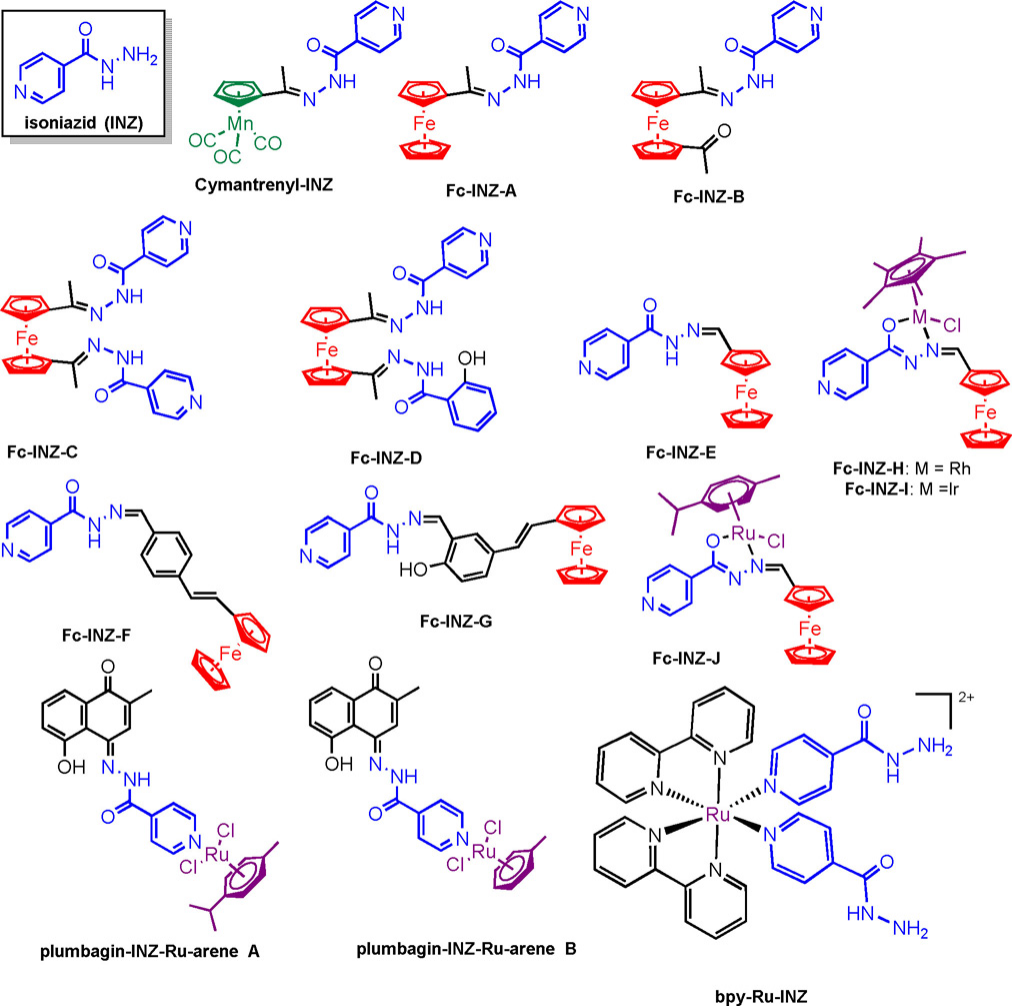
Mn, Fe and Ru organometallic and Ru bipyridyl complexes containing the antibacterial drug isoniazid.^80^, ^81^, ^82^

Organometallic derivatives of the anti‐TB drug isoniazid^78^, ^79^ (**INZ**) have been widely studied, with its pyridyl ring being an attractive metal coordination site. Tirkey et al. reported the synthesis of cymantrenyl hydrazone (**cymantrenyl‐INZ**) and ferrocenyl hydrazone (**ferrocene‐INZ‐A‐D**) analogues of **INZ** (Figure 7).^80^ Selected complexes were tested for activity in bacterial strains *B. subtilis, E. coli, S. aureus, K. pneumoniae* and *P. aeruginosa*. **Cymantrenyl‐INZ** was generally less active (MIC values 125–250 μg mL^−1^) compared to the ferrocenyl analogue (**ferrocene‐INZ‐A**; MIC values: 31.25–125 μg mL^−1^). No data for **INZ** were reported. The activity of these complexes was also compared to reported MIC values for organic derivatives or the ferrocenyl and cymantrenyl complexes; the organometallic complexes were found to have superior antimicrobial activity.^80^ The authors speculated that the presence of the ferrocenyl and cymantrenyl groups may increase cell permeability and lipophilicity of these molecules thus increasing activity. They also suggested that π‐electron delocalisation and hindrance of metal binding sites in crucial enzymes in the microorganisms could also play a role. However, no experimental data confirming this were reported.

Stringer and co‐workers carried out structure–activity studies of ferrocenyl (**ferrocene‐INZ‐E‐G**) and heterobimetallic complexes (**ferrocene‐INZ‐H‐J**) in malarial, trichomonal and mycobacterial strains.^81^ Most of the complexes showed higher antimycobacterial activity towards *M. tuberculosis* (H37Rv strain) in the glycerol‐based GAST‐Fe growth medium (MIC_90_ or MIC_99_<1 μm). **Ferrocene‐INZ‐E** (containing no aromatic spacer between **INZ** and ferrocene) and the heterobimetallic complexes showed activities comparable to the free parent drug, **INZ**. None of the complexes were as active as **INZ** against *Mtb*. As antiparasitics, low to moderate inhibition was observed against the *T. vaginalis* strain G3. Interesting trends were noted in the NF54 chloroquine‐sensitive strain of *P. falciparum*. In contrast to the antimycobacterial activity, **INZ** was found to be inactive against the NF54 *Pf* strain. The **ferrocene‐INZ‐E** was not active (>100 μm) while **ferrocene‐INZ‐F** and **G** containing the aromatic spacer were remarkably more active; ca. 3 times greater for **ferrocene‐INZ‐F** (31.52 μm) and 65 times greater for **ferrocene‐INZ‐G** (1.58 μm). The heterobimetallic complexes **ferrocene‐INZ‐H‐J** also displayed low IC_50_ values of 2.99–7.82 μm. Screening against the normal Chinese Hamster Ovarian (CHO) cell line revealed low toxicity, indicative of good selectively for microbial strains.

Ruthenium‐arene complexes (**plumbagin‐INZ Ruthenium arene A** and **B**) of a hybrid ligand prepared from the natural plant product plumbagin, a yellow naphthoquinone dye, and **INZ** have been reported by Spoerlein‐Guettler et al.^82^ Due to the relative insolubility of **plumbagin‐INZ Ruthenium arene B**, only **plumbagin‐INZ Ruthenium arene A** was tested for anticancer activity on two cell lines that are sensitive to plumbagin (518A2 melanoma and HCT‐116 colon carcinoma cells), the multi‐drug resistant KBV1/VbI cervix carcinoma cell line and non‐malignant CHF chicken heart fibroblast cell line. Compared to free plumbagin and cisplatin, the ruthenium complex showed higher activity in all tumour cell lines and was not toxic to the CHF cell line up to 50 μm. Notably, in the multidrug resistant cell line (KBV1/VbI) **plumbagin‐INZ‐Ru‐arene A** displayed an IC_50_ of 3.80 μm while cisplatin and plumbagin were less active (IC_50_ 11.4 and 26.2 μm, respectively), promising for further work on drugs that can overcome resistance.

Isoniazid has also been incorporated into the Ru^II^ complex **bpy‐Ru‐INZ** (Figure 7). This complex was screened against Gram‐positive *Bacillus subtilis,* Gram‐negative *E. coli*, and *Mycobacterium smegmatis*, which possess the three major classes of cell envelope. It was inactive in the dark, but rapidly (<500 ps) released isoniazid on irradiation of the complex with visible light.^83^ The complex was 6 times more potent towards *M. smegmatis* compared to isoniazid alone and relatively non‐toxic towards normal human cells. This strategy for combatting AMR could be applied to a range of Ru^II^ and Ir^III^ photoactivatable organometallic complexes.

Biguanides can exhibit antimicrobial and antiviral activity, and recently have been incorporated as chelating ligands into Cp*, Cp^xPh^ and Cp^xPhPh^ complexes.^84^ Several such complexes, containing inactive biguanides, exhibit potent activity against Gram‐negative and Gram‐positive bacteria (including methicillin‐resistant *Staphylococcus aureus* (MRSA), and high antifungal potency towards *C. albicans* and *C. neoformans*, with minimum inhibitory concentrations (MICs) in the nanomolar range, and low cytotoxicity towards mammalian cells. They can restore the activity of vancomycin against vancomycin‐resistant *Enterococci* (VRE) and disrupt and eradicate bacteria in mature biofilms.

## Derivatives of Antiparasitic Drugs

**Figure 8:**
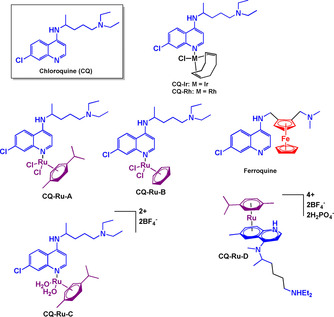
Structures of chloroquine, ferroquine and ruthenium arene chloroquine conjugates.^6^, ^93^, ^94^

The aminoquinoline pharmacophore has been incorporated into many antiparasitic drugs, particularly antimalarials.^85^, ^86^ Although resistance to several of the current quinoline drugs is emerging, they still remain attractive for derivatisation.^85^ Organometallic conjugates of such drugs also show potential for overcoming resistance, Ferroquine being an important example.^87^, ^88^, ^89^, ^90^ Chloroquine (**CQ**, Figure 8) belongs in the 4‐aminoquinoline structural group and is currently used in regions affected by **CQ**‐sensitive strains of *P. vivax*, *P. ovale*, and *P. malariae*. It is no longer recommended for treatment of malaria caused by *P. falciparum*, due to extensive resistance.^91^ **CQ** is administered orally and has also been prescribed for other illnesses such as rheumatoid arthritis and lupus.^92^

**Figure 9:**
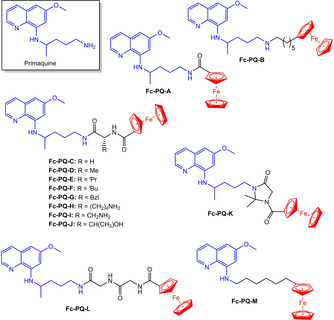
Structures of primaquine and ferrocene–primaquine conjugates.^92^, ^95^, ^96^

The mononuclear Rh^I^ complex (**CQ‐Rh**) proved active against *P. falciparum* strains in vitro.^93^ It exhibits activity similar to **CQ** diphosphate, and in vivo studies against rodent malaria parasite *P. berghei* revealed that at the same concentration of **CQ** diphosphate required to kill 50 % of parasitemia, **CQ‐Rh** reduced parasitemia by 74 %. Ferroquine has a ferrocene moiety incorporated into the side‐chain of **CQ** and has impressive antiplasmodial activity in both **CQ**‐sensitive and **CQ**‐resistant strains.^6^, ^88^, ^89^, ^90^ It has completed Phase I and is expected to complete Phase II clinical trials against uncomplicated malaria soon. Ruthenium‐arene **CQ** conjugates (**CQ‐Ru‐A–D**) have been evaluated for in vitro activity against three chloroquine resistant (W2, Dd2 and K1) and four chloroquine sensitive (FcB1, 3D7, PFB and F32) *P. falciparum* strains.^94^ They were moderate inhibitors of the chloroquine‐sensitive *P. falciparum* strains with activities lower than **CQ** diphosphate. However, against **CQ**‐resistant strains, these complexes displayed consistently better activity compared to **CQ** diphosphate. The most potent inhibitor was **CQ‐Ru‐C** (Figure 9), which was 5x more potent than **CQ** disphosphate against the Dd2 and K1 strains. These results illustrate clearly the concept of enhanced activity of metal‐chloroquine conjugates against resistant malarial strains.

The antimalarial primaquine (**PQ**, Figure 9) is a prescribed oral treatment for malaria caused by *P. vivax* and *P. ovale*, and *Pneumocystis pneumonia*.^95^ Matos et al have studied the antimicrobial efficacy towards plasmodial, leishmanial and pneumocystal strains of a library of ferrocene‐primaquine conjugates (**Fc‐PQ‐A–M**).^92^, ^95^, ^96^

Against the CQ‐resistant *P. falciparum* strain W2, only complex **Fc‐PQ‐F** was active, whilst all of them were active against *Pneumocyctis carinii*.^92^ The complexes containing amino acid type linkages between **PQ** and ferrocene showed anti‐Pneumocystis activity with **Fc‐PQ‐F** and **Fc‐PQ‐G** being the most potent (inhibitory concentrations; 2.69 and 1.15 μg mL^−1^, respectively). It appears that the degree of lipophilicity of the amino acid linkers in these complexes may play a role; higher activity was observed for the complexes that contained the more lipophilic groups (iso‐butyl and benzyl). *In vivo* studies of the complexes in the liver stage model *P. berghei* ANKA‐GFP‐infected BALB/c mice and *Anopheles stephensi* mosquitoes were carried out to confirm if these complexes could inhibit the sporogonic cycle of plasmodia.^96^ Complexes **Fc‐PQ‐B** and **Fc‐PQ‐G** displayed high dose‐dependent inhibition with the former able to completely impair the parasite's sporogonic cycle at 50 μmol kg^−1^ (95 % inhibition); a significantly higher activity than free PQ (26.9 % inhibition). In vitro *P. berghei* inhibition assays found that most of the complexes show low micromolar IC_50_ values with five of the **FC‐PQ** complexes performing better (IC_50_ 0.17–2.82 μm) than PQ (IC_50_=7.50 μm). As a result of the appreciable antiplasmodial activity, they were also screened against the promastigote and intramacrophagic amastigote forms of *Leishmania infantum*.^95^ All complexes inhibited growth of *L. infantum* to some degree and complex **Fc‐PQ‐C** was identified as a potential new antileishmanial lead. At 60 and 40 μm concentrations, it caused >96 % reduction in amastigotes per 100 macrophages in the intramacrophagic amastigote form of the parasite.

**Figure 10:**
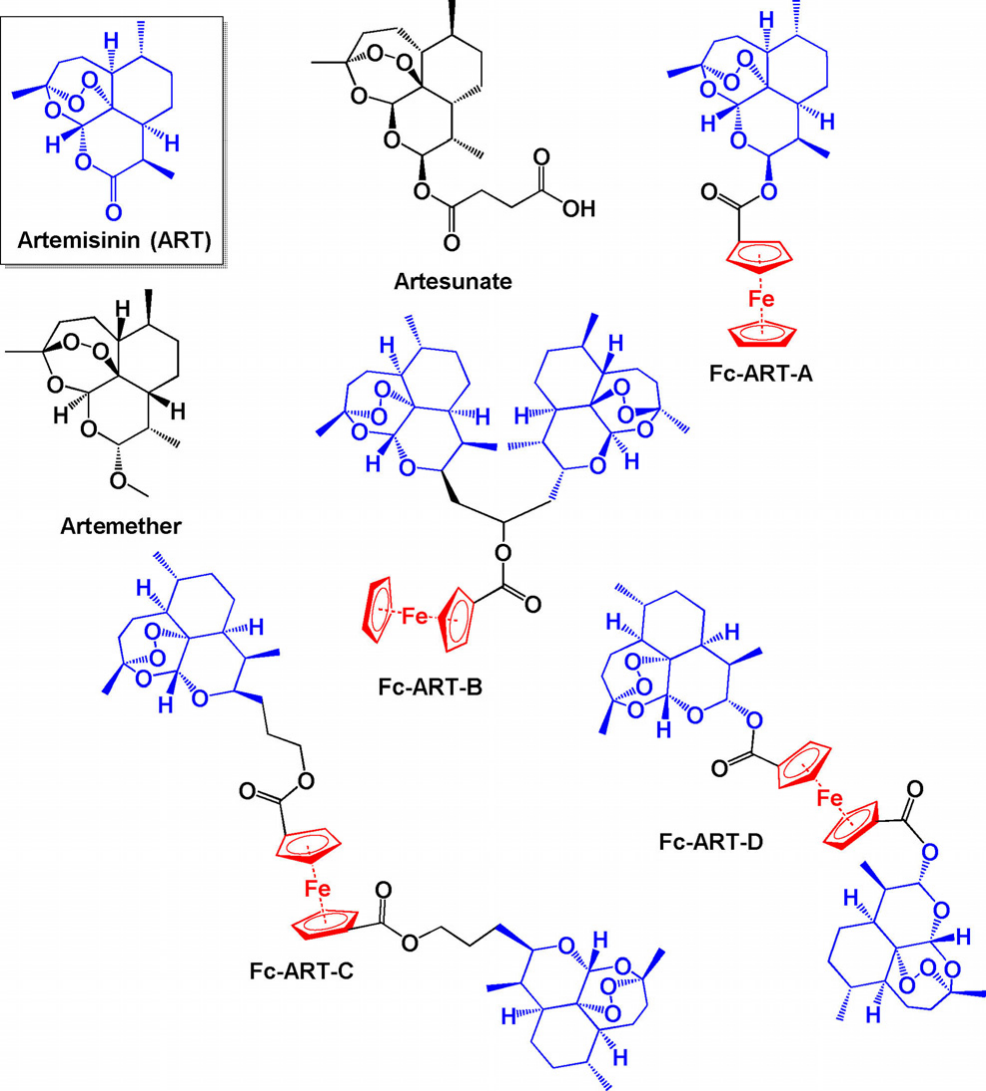
Ferrocenyl artemisinin derivatives **Fc‐ART‐A‐D**.^100^

Artemisinin is a natural product that has been used for decades to treat malaria. Its derivatives, artesunate and artemether (Figure 10) are also part of the arsenal of WHO‐recommended drugs for malarial infections caused by *P. falciparum*.^97^ The mechanism of action of artemisinin (**ART**) and its derivatives has yet to be clearly defined, but it is accepted that these drugs selectively target parasites because of their enhanced uptake by parasitized erythrocytes compared to healthy erythrocytes.^98^ The endoperoxide bridge present in ART likely interacts with the iron in haem to generate radical species that affect the redox balance of the malaria parasite.^99^

**Figure 11:**
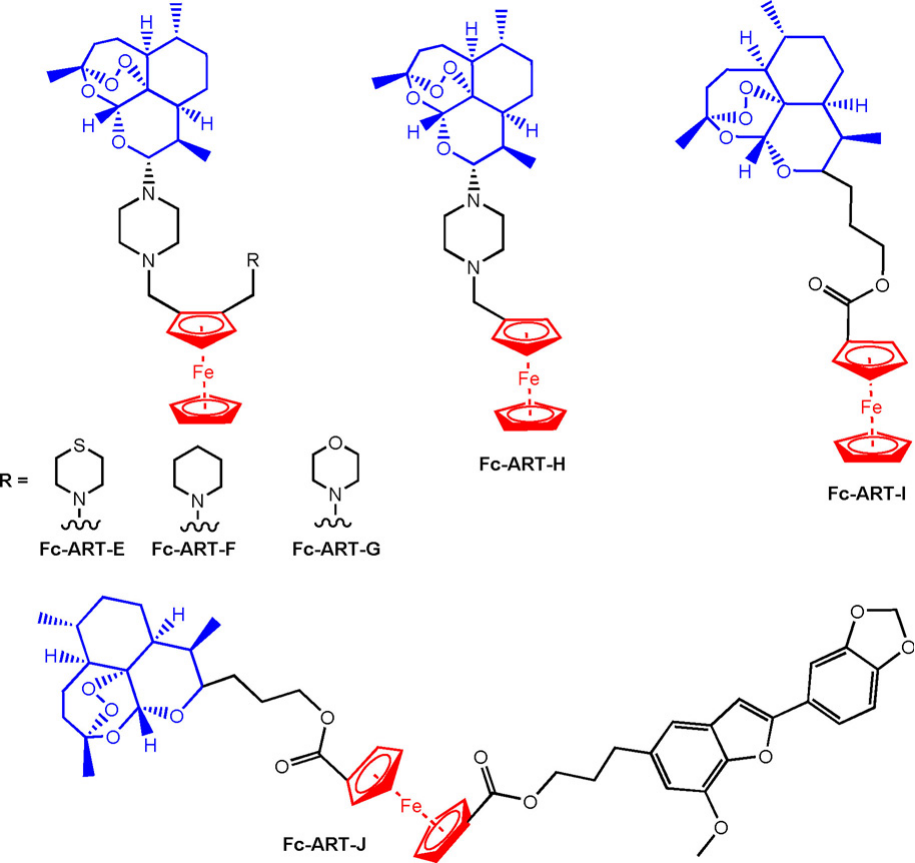
Ferrocenyl‐artemisinin hybrids **Fc‐ART‐E‐J**.^101^, ^102^

Reiter and co‐workers synthesised a series of ferrocene‐artemisinin (**Fc‐ART**) derivatives (Figure 10) that contained one or two ART moieties.^100^ Their activities were assessed against two leukaemia cell lines (human acute lymphoblastic CCRF‐CEM and the multidrug resistant CEM/ADR5000), human cytomegalovirus (HCMV) and *Pf* strain 3D7. All the complexes (**Fc‐ART‐A–D**) were highly potent against CCRF‐CEM cells (IC_50_≤0.13 μm) and CEM/ADR5000 (IC_50_≤1.96 μm). The complexes containing two ART moieties (**Fc‐ART‐B** and **Fc‐ART‐D**) are almost twice as active against CCRF‐CEM cells as the complexes with just one ART (**Fc‐ART‐A**). However, for CEM/ADR5000, the opposite effect was observed. With the exception of **Fc‐ART‐C**, all the complexes exhibited activities of <0.50 μm towards the HCMV cell line. Their inhibitory effect on the CQ‐sensitive *P. falciparum* strain was also significant (IC_50_ values 7.20–30.2 nm).

de Lange et al recently reported a series of amino‐artemisinin‐1,2‐disubstituted ferrocene hybrids which contain a piperazine linker between ART and various ferrocenyl moieties (**Fc‐ART‐E‐H**, Figure 11).^101^ They were tested on asexual blood stage strains of *P. falciparum* (**CQ**‐sensitive NF54 and **CQ**‐resistant K1 and W2) as well as early and late blood stage gametocytes (ESG‐NF54 and LSG‐NF54). All complexes displayed notable activity, with **Fc‐ART‐G**, which contains a morpholino fragment bonded to Fc, being the most potent (IC_50_=0.86 nm (K1) and 1.4 nm (W2)). Gametocytocidal activity was tested at 1 μm and 100 nm concentrations and for ESG‐NF54 complexes **Fc‐ART‐E**‐**G** showed ≥93 % inhibition at both concentrations; they were slightly less active (≥84 %) in LSG‐NF54. **Fc‐ART‐E**‐**H** were further screened against the human melanoma cell line A375 and normal human embryonic kidney cell line HEK293. With the exception of **Fc‐ART‐G**, all complexes had higher activity against A375 compared to HEK293. It is significant that ferrocenyl‐artemisinin derivatives inhibited both sexual and asexual blood stages of *P. falciparum*, since this may lead to the preparation of new drugs that can block transmission.

Complexes **Fc‐ART‐I** and **Fc‐ART‐J** also have high antiproliferative activity towards CCRF‐CEM lymphoblastic leukaemia cells.^102^ **Fc‐ART‐J** contains a ferrocenyl diester bridge between the ART moiety and the bioactive natural benzofuran eugonol and was less potent than **Fc‐ART‐I** towards drug‐resistant CEM/ADR5000. Overall, **Fc‐ART‐I** is a promising new lead with good efficacy in both leukaemia cell lines.

Recently, Gasser et al. have investigated the design of organometallic derivatives of various anthelmintic drugs as anti‐parasitic agents.^103^, ^104^, ^105^, ^106^, ^107^ Anthelmintics are a class of drugs used to treat diseases caused by parasitic worms. One of their earliest reports focussed on ferrocenyl derivatives of Praziquantel (**PZQ**).^103^ **PZQ**, marketed as Biltricide, an effective treatment for urogenital and intestinal schistomiasis.^108^ However, low cure rates have been reported in some countries thus prompting study of new drug leads.

**Figure 12:**
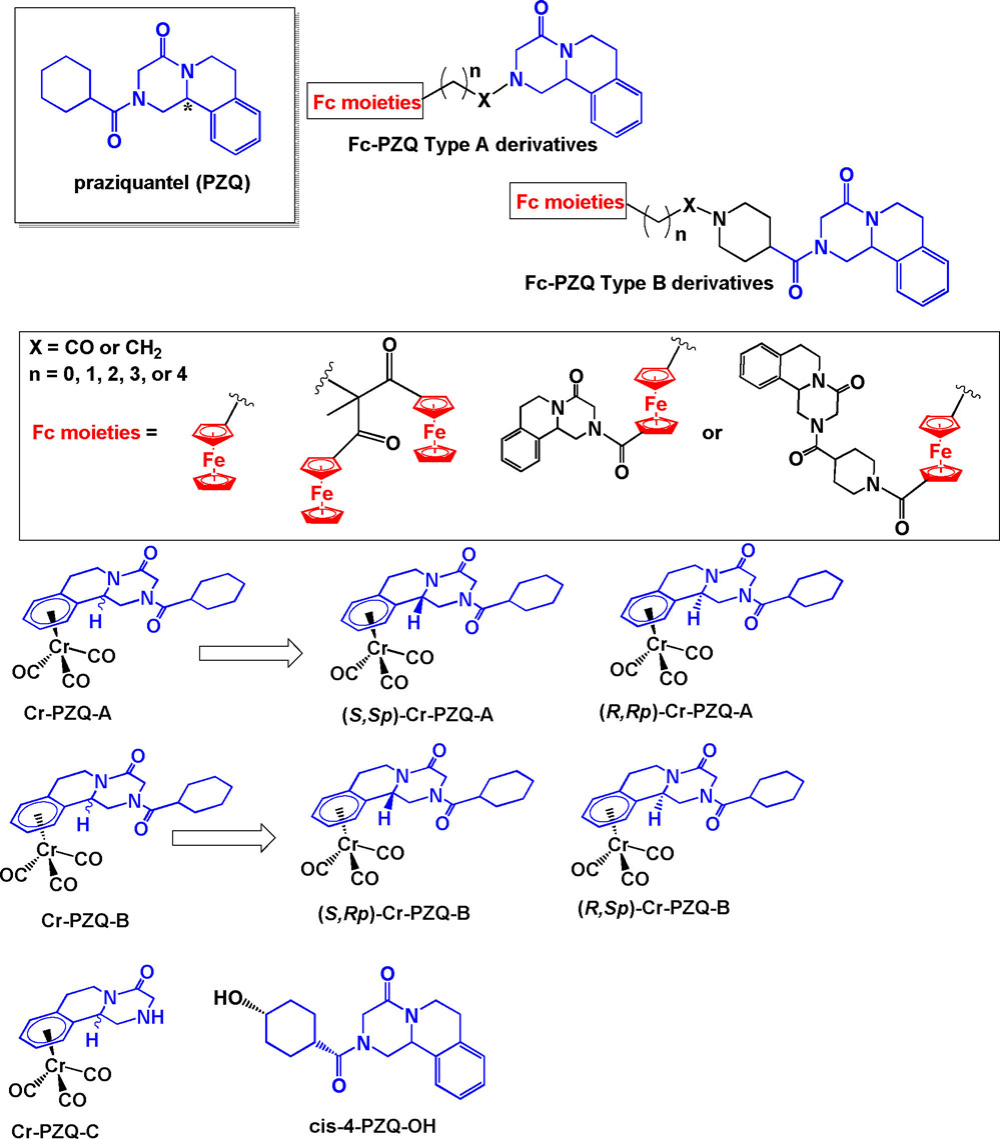
Structures of **PZQ** and its ferrocenyl and chromium‐carbonyl derivatives.^103^, ^104^, ^107^

An extensive library of **Fc‐PZQ** complexes (Figure 12) was tested for activity on *Schistosoma mansoni* (*S. mansoni*), cervical cancer cell line HeLa and the healthy lung cell line MRC‐5. Most of the complexes were either inactive or mildly active against *S. mansoni*. The **Fc‐PZQ** derivatives that showed some inhibitory effects on HeLa cells were less active in healthy MRC‐5 cells.^103^ Half‐sandwich chromium carbonyl complexes of PZQ were also prepared.^104^, ^107^ PZQ has a stereogenic centre (labelled * in Figure 12), and thus the complexes were initially tested as racemic mixtures (**Cr‐PZQ‐A** and **Cr‐PZQ‐B**).^107^ Compared to their ferrocenyl analogues, **Cr‐PZQ‐A** and **Cr‐PZQ‐B** were better inhibitors of *S. mansoni*, displaying IC_50_ values of 0.25 and 0.27 μm respectively. Additionally, they were inactive up to a concentration of 100 μm against MRC‐5 cells and showed low activity in HeLa cells. The complexes were also relatively stable in D_2_O and in human plasma. These promising results prompted investigation into preparation and testing of the pure optically active diastereomers of **Cr‐PZQ‐A** and **Cr‐PZQ‐B** and the three major metabolites identified from in vitro metabolism studies (**PZQ**, **Cr‐PZQ‐C** and ***cis*** **‐4‐PZQ‐OH**, Figure 11).^104^ The data showed that only the (*R*)‐isomers, complexes **(*R,Rp*)‐Cr‐PZQ‐A** and **(*R,Sp*)‐Cr‐PZQ‐B**, were highly active with IC_50_ values similar to free **PZQ**. They were also better inhibitors compared to **Cr‐PZQ‐C** and ***cis*** **‐4‐PZQ‐OH**. *In vivo* studies in mice infected with chronic *S. mansoni* revealed that, in contrast to the in vitro data, the racemates **Cr‐PZQ‐A** and **Cr‐PZQ‐B** were toxic and only reduced the worm burden by 24 and 29 %, respectively, at a single dose of 400 mg kg^−1^. At this dose, **PZQ** reduces the worm burden by 96 %.

**Figure 13:**
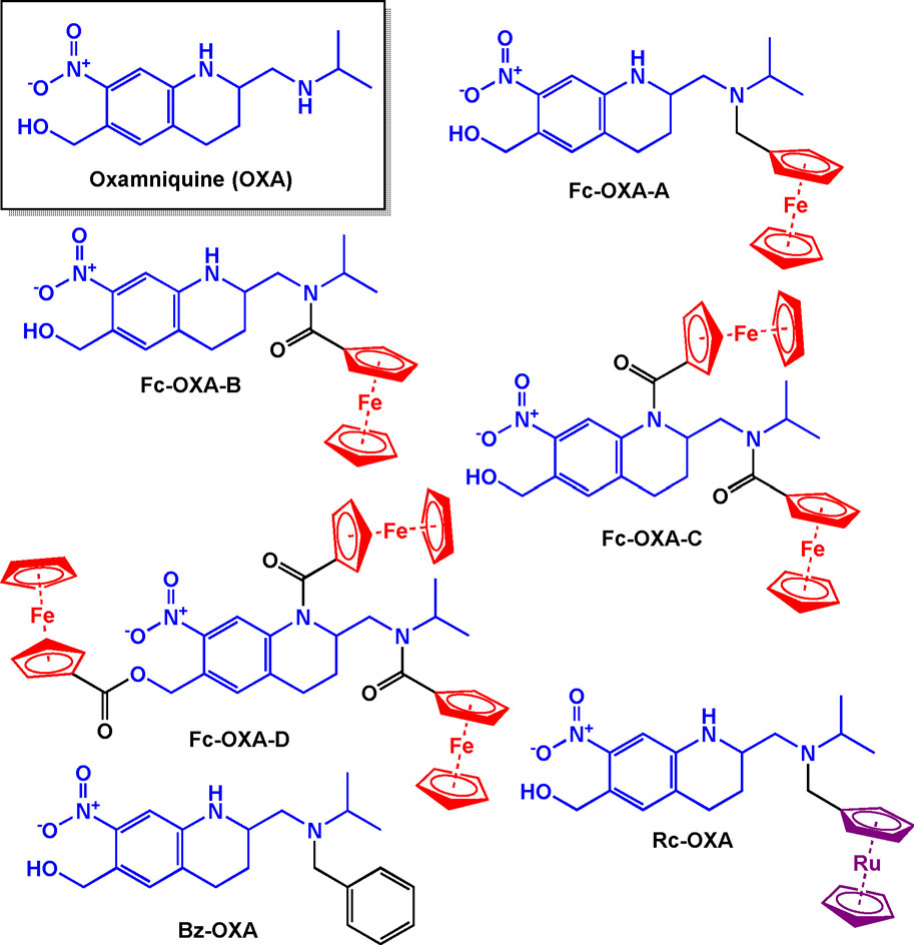
Structures of **OXA**, its ferrocenyl and ruthenocenyl derivatives.^105^

Oxamniquine (**OXA**) is another antischistosomial agent and organometallic derivatives of **OXA** (Figure 13) were designed with the premise that these complexes may show different binding modes compared to free **OXA** in the enzyme, *S. mansoni* sulfotransferase.^105^ The most active compounds in vitro were also tested in an in vivo mouse model. Of the four ferrocenyl derivatives **(Fc‐OXA‐A‐D**), only **Fc‐OXA‐A** showed 100 % reduction in the viability of *S. mansoni* newly transformed schistosomula (NTS) in vitro. **Fc‐OXA‐B** was the second most active complex at 76.2 %; higher than free **OXA**. In the in vivo studies, at a 200 mg kg^−1^ dose, only **Fc‐OZA‐A** and free **OXA** showed 100 % clearance of all worms from the host.

Two additional **OXA** derivatives, **Rc‐OXA** and **Bz‐OXA** were synthesised to understand whether the ferrocene moiety of **Fc‐OXA‐A** is essential for activity, or if a purely organic derivative would be more active. In both the in vitro and in vivo models, the ruthenocenyl complex (**Rc‐OXA**) showed superior activity over **Fc‐OXA‐A** and **Bz‐OXA**.

**Figure 14:**
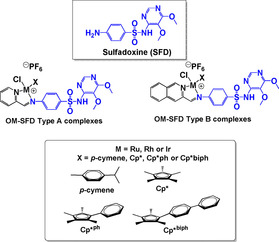
Structures of Sulfadoxine and some precious metal organometallic half‐sandwich conjugates.^109^

Organo‐ruthenium, rhodium and iridium derivatives of the sulfa‐drug sulfadoxine (Figure 14) have been reported by Chellan et al.^109^

Screening for in vitro activity against CQ‐sensitive 3D7, CQ‐resistant Dd2 and LSGNF54 parasite strains and *Mycobacterium tuberculosis* found that the **OM‐SFD** ruthenium derivatives were inactive. The Rh analogues were more potent than their iridium counterparts but overall, the **OM‐SFD type B** iridium complex containing the quinoline‐imino functionality and the Cp^xbiph^ ligand was the most active in the whole library. Moreover, the parent drug, sulfadoxine, was not active in most of the assays, affirming the hypothesis that organometallic conjugates of drugs can beneficially affect bioactivity. Most of the Rh and Ir complexes displayed inhibitory activity in the sexual LSG assay, while the clinical drugs, pyrimethamine, sulfadoxine and chloroquine were not. This is yet another study that validates the idea of organometallic conjugation of a drug can alter its biological target, as noted by others.^110^, ^111^, ^112^, ^113^, ^114^, ^115^, ^116^, ^117^, ^118^, ^119^, ^120^, ^121^, ^122^

## Derivatives of Antifungal Drugs

**Figure 15:**
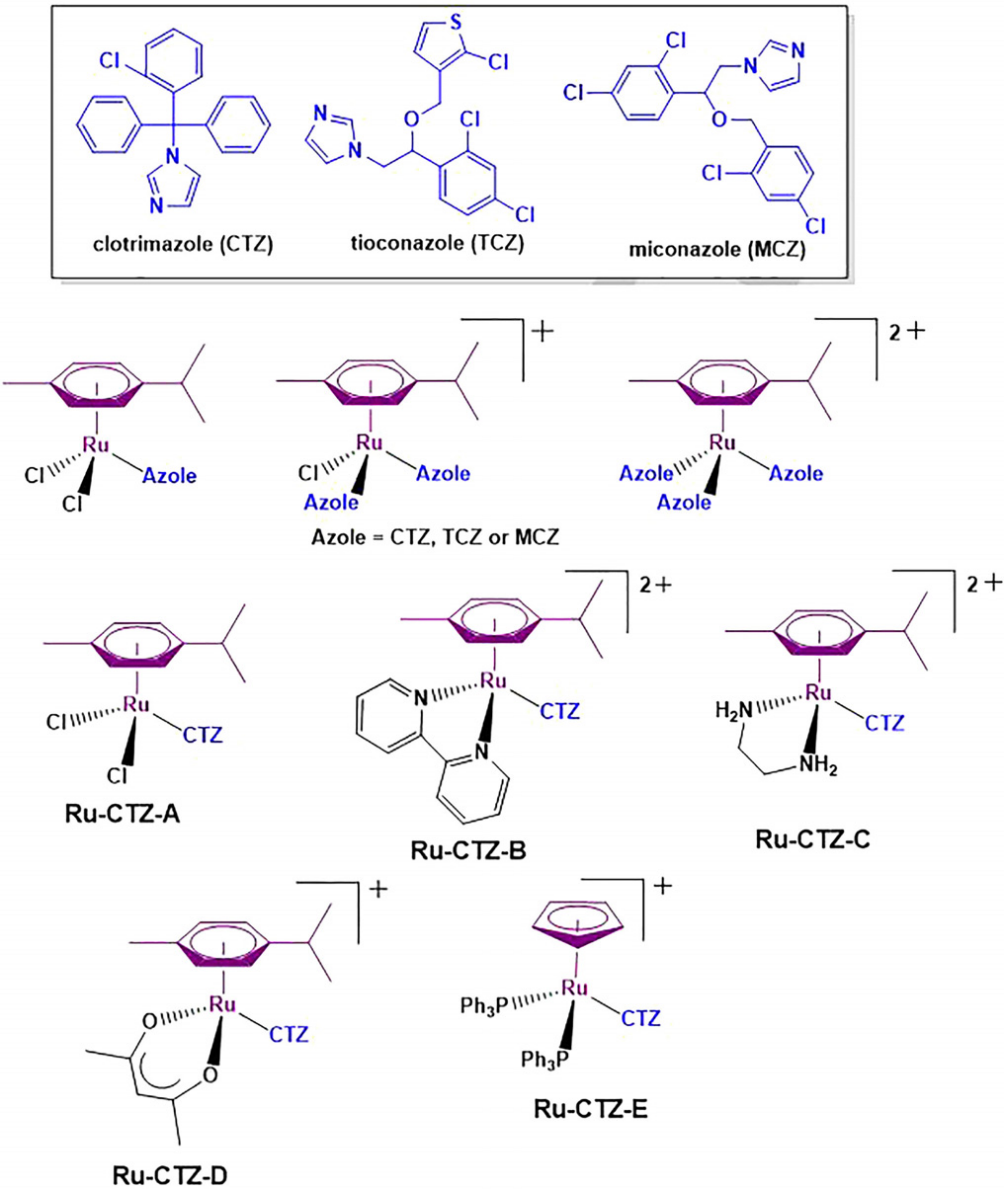
Structures of **CTZ**, **MCZ**, **TCZ** and their respective ruthenium‐arene or ruthenium‐cyclopentadienyl complexes.^123^, ^124^, ^125^

Clotrimazole (**CTZ**), miconazole (**MCZ**) and tioconazole (**TCZ**) (Figure 15) belong to the azole antifungal drug class. Mono‐, bis‐ and tris‐azole ruthenium‐arene complexes, where the azole drug is bound to the metal via one of the imidazole nitrogen atoms, were assayed for antifungal and antiparasitic activity.^123^ As antifungals, none of the complexes were as active as their corresponding free azole drug. However, the complexes showed good reduction in radial fungal growth at both 0.5 and 0.01 mm concentrations. At a dose of 100 μg mL^−1^, all of the complexes containing **MCZ** as ligand(s) showed moderate antischistosomal activity, and interestingly, the tris‐**MCZ** ruthenium complex also showed gender‐specific selectivity. After 72 h exposure to the complex, female worms showed only a marginal decrease in motility, while all of the male worms died. The mono‐azole complexes decomposed slightly in DMSO, while the bis‐ and tris‐ complexes remained intact.

Sanchez‐Delgado and co‐workers prepared a small library of ruthenium‐arene CTZ complexes (**Ru‐CTZ‐A‐D**) for application as antileishmanial and antitrypanosomal agents.^124^ They noted a synergistic effect on activity of **CTZ** metalation. All of the complexes displayed LD_50_ values lower than free CTZ for both promastigotes of *L. major* and epimastigotes of *T. cruzi*. The most potent complex was Ru‐CTZ‐A (LD_50_=0.015 μm (*L. major*) and 0.10 μm (*T. cruzi*)); respectively 110× and 58× more active than free **CTZ. Ru‐CTZ‐A‐D** also did not show any appreciable toxicity towards healthy human osteoblast cells.

The cyclopentadienyl Ru complex, **Ru‐CTZ‐E**, has more potent antiparasitic and antitumor activities compared to free **CTZ**.^125^ In *T. cruzi* and *T. brucei*, **Ru‐CTZ‐E** was 7× (IC_50_=0.25 μm) and 41× (IC_50_=0.60 μm) more active than **CTZ**, suggesting that this complex could be developed as a broad spectrum drug for trypanosomatid parasites. It was also highly cytotoxic in ovarian A2780, breast MCF7, and cervical HeLa cancer cell lines, inhibiting proliferation at concentrations between 0.50 and 5.0 μm.

Compared to free **CTZ** (IC_50_=5.7–20.5 μm) and cisplatin (IC_50_=1.9–28 μm), **Ru‐CTZ‐E** was a more potent antitumor agent, suggesting potential for anticancer treatment.

## Conclusions

In this review we have described briefly how the conjugation or direct coordination of clinical drugs to organometallic fragments can enhance or restore biological activity of anticancer and antimicrobial drugs. Such conjugation has an effect on drug uptake and delivery and can change target sites, so introducing modified mechanisms of action that can combat resistance, and sometimes reduce off‐target toxicity,^51^, ^52^, ^53^, ^54^

The best studied anticancer conjugates are the ferrocene‐tamoxifen derivatives of Jaouen et al. which target oestrogen receptors.^57^ The addition of the ferrocene fragment introduces new redox chemistry into the mechanism of action through formation of quinone methides as a result of oxidative metabolism. Although structurally analogous, the ruthenocene and osmocene derivatives have different redox properties and are less active. Similarly, organometallic conjugation can improve the activity of antibiotics. The most successful example to date is that of ferroquine which as a chloroquine‐ferrocene conjugate is active against resistant malarial strains, and relatively non‐toxic (at gram doses).^87^ Ferroquine is not only in clinical trials for malaria treatment, but also a candidate for repositioning as a cancer therapeutic.^126^ Its ability to induce lysosomal dysfunction may be important to the mechanism of anticancer activity. Ferrocenyl conjugates of the antimalarial drug artemisinin also exhibit antiparasitic activity, and interestingly also exhibit antileukaemic activity. Organometallic half‐sandwich fragments can also be effective in restoring activity to antibiotics. For example, some cyclopentadienyl Ir^III^ fragments can restore activity to the antiparasitic drug sulfadoxine.^109^

Fragment‐based drug design (FBDD) is now a well‐established method for exploring chemical space and discovering new drugs.^127^ Several drugs designed using this approach have already entered the clinic. The examples described here show that organometallic fragments can bring a new dimension to FBDD, providing fragments which offer more than just structural (e.g. H‐bonding, hydrophobic and electrostatic) interactions with target sites such as proteins. Organometallic fragments offer redox centres with potentials tunable by not only the C‐bound ligand(s), but also the other ligands on the metal ion. They can also act as transporters of other biologically active molecules. In transition metal complexes, C‐bound ligands often have high *trans* (and *cis*) effects which promote the release of other ligands. Organometallic fragments containing carbon monoxide ligands, for example, can be activated by photo‐irradiation,^128^ a potentially attractive procedure for achieving selective attack on target cells and minimizing side‐effects. Bearing in mind the large number of metal ions which form stable organometallic compounds, the range metal oxidation states available, as well as variations in coordination number and geometry, organometallic fragments can introduce a vast range of new pharmacophores with unique interactions with target sites. This strategy holds much promise for the discovery of drugs with novel mechanisms of action which can combat resistance.

## Conflict of interest

The authors declare no conflict of interest.

## Biographical Information

*Prinessa Chellan received her PhD from the University of Cape Town in 2013. She followed this with a postdoctoral position in the laboratory of Prof. Peter J. Sadler at the University of Warwick (2014–2016) where she worked on metal conjugates of antimalarial drugs. She is now a lecturer in the Department of Chemistry and Polymer Science at Stellenbosch University. Her current research is focused on the design and study of novel organometallic complexes for different illnesses including, cancer and malaria*.

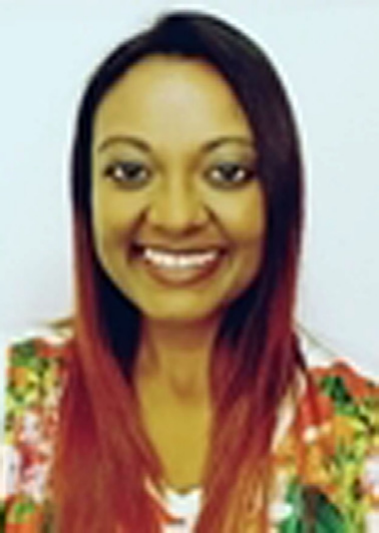

## Biographical Information

*Peter Sadler obtained his BA, MA and DPhil at the University of Oxford. Subsequently he was a MRC Research Fellow at the University of Cambridge and National Institute for Medical Research. From 1973–96 he was Lecturer, Reader and Professor at Birkbeck College, University of London, and from 1996–2007 Crum Brown Chair of Chemistry, University of Edinburgh. Then he became Head of the Department of Chemistry at the University of Warwick, where he is now a Professor. He is a Fellow of the Royal Society of Chemistry (FRSC), Royal Society of Edinburgh (FRSE), the Royal Society of London (FRS), and the European Academy of Sciences, Honorary Fellow of the Chemical Research Society of India and Chinese Chemical Society, and an EPSRC RISE Fellow (Recognising Inspirational Scientists and Engineers)*.